# When macrophages have more than one thing to show

**DOI:** 10.1111/bjh.70618

**Published:** 2026-07-07

**Authors:** Hachem Abou Moustafa, Maïlys Le Guyader, Pierre Loiseau, Yohann Le Govic, Thomas Boyer

**Affiliations:** ^1^ Service d'Hématologie Biologique CHU Amiens‐Picardie Amiens France; ^2^ Service de Médecine Interne CHU Amiens‐Picardie Amiens France; ^3^ Service de Parasitologie‐Mycologie CHU Amiens‐Picardie Amiens France

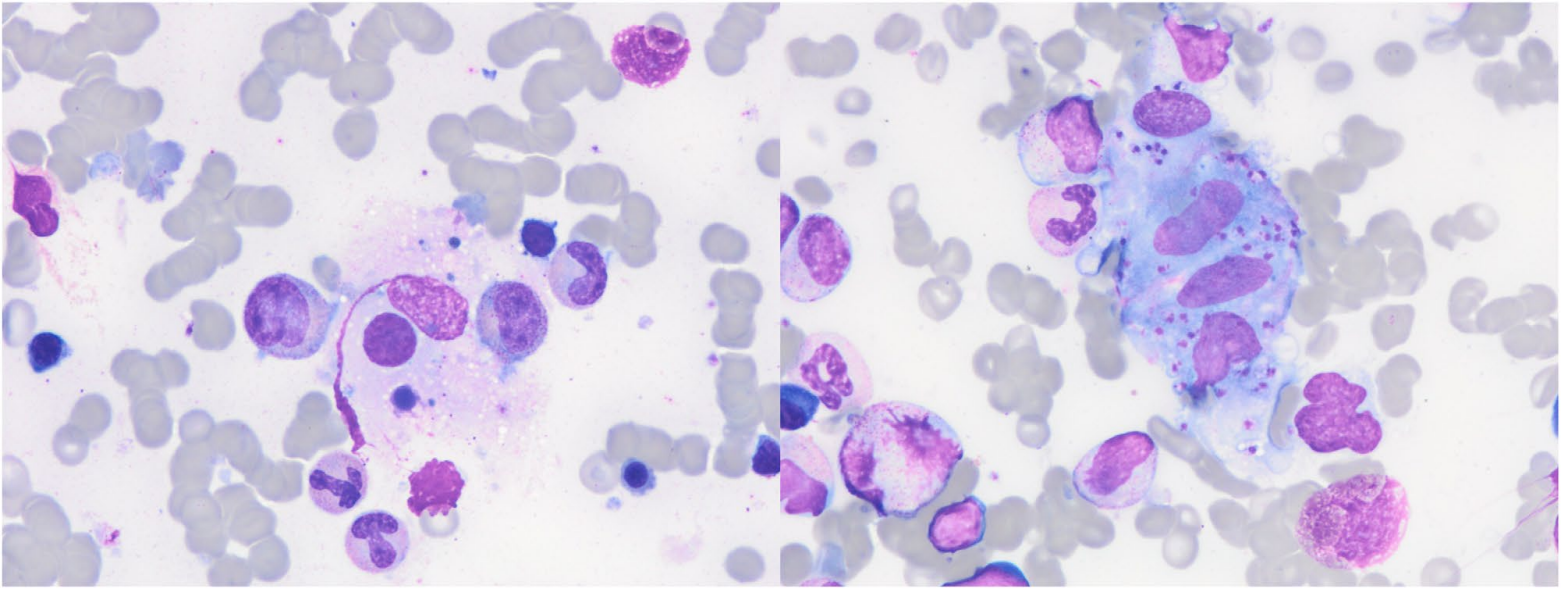

A 31‐year‐old man with no prior medical history was hospitalised in the internal medicine department for asthenia, fluctuating pyrexia at 39–40°C for 3 weeks, night sweats and anorexia (10 kg weight loss in 1 month). Computed tomography of the thorax, abdomen and pelvis imaging revealed hepatosplenomegaly. He reported a stay in Ardèche (France) 15 months before the onset of symptoms. A full blood count revealed pancytopenia: leucocyte count of 1.7 × 10^9^/L (with no hypereosinophilia), haemoglobin concentration of 81 g/L and platelet count of 67 × 10^9^/L. Due to the persistence of cytopenias, a bone marrow aspiration was performed, showing signs of haemophagocytosis (left, May–Grünwald–Giemsa staining, objective × 100) and Leishman–Donovan bodies (right, May–Grünwald–Giemsa staining, objective × 100). The diagnosis of visceral leishmaniasis (VL), later supported by polymerase chain reaction (PCR), associated with macrophage activation syndrome (MAS), as the patient fulfilled several diagnostic criteria including fever, organomegaly, pancytopenia, hyperferritinaemia, hypertriglyceridaemia, hypofibrinogenaemia and elevated aspartate aminotransferase (AST).^1^ For VL, treatment with liposomal amphothericin B was initiated for 5 days, in combination with hydrocortisone for MAS. At the 1‐month follow‐up, a dramatic improvement of clinical and biological parameters was observed, with apyrexia, disappearance of hepatosplenomegaly and normalisation of the blood count.

MAS or haemophagocytic lymphohistiocytosis is a rare but life‐threatening syndrome. It can arise from genetic disorders (rare) or secondary causes such as infection (mostly viral), autoimmunity or malignancy. The pathophysiological mechanism of MAS is thought to be based on a major dysregulation of the immune response, marked by excessive and uncontrolled production of pro‐inflammatory mediators, responsible for systemic inflammation and multiorgan failure. Identifying the underlying trigger of a MAS episode is essential, as adjunctive therapies in the acute setting may vary depending on associated disorders. VL is a rare cause of MAS and remains a diagnostic challenge due to similar clinical presentations; however, the frequency of MAS in patients diagnosed with VL is high, at around 40%.^2^ Physicians should consider the diagnosis of VL in patients presenting with fever and pancytopenia who have travelled to a department located in an endemic area, as in the present case.

## FUNDING INFORMATION

The authors have nothing to report.

## ETHICS STATEMENT

The authors have nothing to report.

## PATIENT CONSENT STATEMENT

The patient's consent has been obtained.

## References

[1] Fardet L , Galicier L , Lambotte O , Marzac C , Aumont C , Chahwan D , et al. Development and validation of the H Score, a score for the diagnosis of reactive hemophagocytic syndrome. Arthritis Rheumatol. 2014;66(9):2613–2620. 10.1002/art.38690 24782338

[2] Horrillo L , Castro A , al Matia B e . Clinical aspects of visceral leishmaniasis caused by *L. infantum* in adults. Ten years of experience of the largest outbeak in Europe: what have we learned? Parasit Vectors. 2019;12(1):359. 10.1186/s13071-019-3628-z 31340851 PMC6657057

